# Frankfurt microsurgery course: the first 175 trainees

**DOI:** 10.1007/s00068-016-0759-1

**Published:** 2017-02-04

**Authors:** G. Perez-Abadia, M. Janko, L. Pindur, M. Sauerbier, J. H. Barker, I. Joshua, I. Marzi, J. Frank

**Affiliations:** 1 Department of Physiology, University of Louisville, Louisville, USA; 2 Department of Trauma, Hand and Reconstructive Surgery, JW Goethe-University, Frankfurt/main, Germany; 3 Frankfurt Initiative for Regenerative Medicine, JW Goethe-University, Frankfurt/main, Germany; 4 Department of Plastic, Hand & Reconstructive Surgery, BG Trauma Center, Frankfurt/main, Germany

**Keywords:** Frankfurt microsurgery course, Microsurgery, Training, Education, Skills, Evaluation in microsurgery

## Abstract

**Introduction:**

Microsurgery courses, taught external to surgical training programs, are essential for acquiring the high level of technical skill required for clinical proficiency.

**Methods:**

The Frankfurt microsurgery course is a 5-day, intensive course that teaches arterial and venous anastomosis using end-to-end, end-to-side, one-way-up, continuous-suture, and vessel graft techniques. During the course, the instructor records the level of skill (in-course data) achieved by each trainee by assessing anastomosis completion and patency. Demographic information is also collected. Post-course trainees are invited to complete an online survey (post-course data) to get their opinions of the courses’ effectiveness.

**Results:**

The in-course “skill achievement” and post-course “course effectiveness” data are presented below. In-course data: 94.8 and 59.9% of participants completed patent end-to-end arterial and venous anastomoses, respectively, while 85.4% performed a patent end-to-side anastomosis. 96.1 and 57.1% of participants who attempted arterial and venous anastomoses using the one-way-up technique were successful, as were 90.9% of those attempting continuous-suture technique. Patent venous grafts were performed by 54.7% of participants.

**Post-course data:**

All respondents indicated significant improvement of their microsurgical skills after taking the course. 66.7% of respondents considered the full-time presence of the instructor to be the most valuable aspect of the course. All respondents would highly recommend the course to colleagues.

**Conclusion:**

The microcourse significantly increased trainees’ clinical microsurgery skills, confidence, and the number of clinical cases they perform. Of all the anastomosis techniques taught, venous anastomosis and grafting were the most difficult to learn. The presence of a full-time experienced instructor was most important.

## Introduction

Microsurgery requires a high level of refined motor skills and is used in several different surgical subspecialties, such as plastic and reconstructive-, hand-, vascular-, neuro-, orthopedics/trauma-, and maxillofacial surgeries and obstetrics and gynecology, ophthalmology, and otolaryngology, as well as in animal research. Microsurgery is a skill that requires fine manual dexterity and like many skills acquired in surgery is primarily taught through the traditional “see one, do one, teach one” method [1]. While this time-tested method has been largely successful for teaching many surgical skills, microsurgery is an exception and requires additional special training to gain clinical proficiency.

Although no standardized curricula for teaching microsurgery is available, to gain the skills needed for clinical proficiency, it is essential that one learns certain basic principles that form a foundation upon which the needed skills can be built. In general, most microsurgery courses consist of a 5-day program, which utilize non-living models at the beginning and progress on to live animal models, usually rats, towards the end [2]. In recent years, there has been a push to introduce more non-living models, which are especially suited for instruction on the use of the microscope, handling micro-instruments and developing basic knot tying skills [3–7]. However, live animal models are preferred, because they more closely simulate clinical microsurgery with real live vessels that bleed, contract, expand, and that have branches that must be ligated to prevent bleeding. In addition, most importantly, live animal vessels form clots at the microsurgical anastomosis, an invaluable indicator of technical proficiency and ultimate success of the anastomosis [8–10].

While the pressure to learn new surgical skills and gain competence continues to grow, access to proper microsurgical training with the necessary expertise of the instructor, and adequate facilities and equipment is not provided in most surgical training programs. To address this deficiency, in Germany, many residents seek extra microsurgical training outside their formal programs. In a survey conducted in 2014 in Germany among plastic surgery residents, 59% indicated that they had attended microsurgery training courses, outside their residency training programs [11]. There are several excellent microsurgery-teaching programs in Germany, both associated with and independent from surgical residency programs. Herein, we present one such program, based at the JW Goethe University in Frankfurt, called the Frankfurt Microsurgery Teaching Course (http://www.microsurgerycourse.de).

**Fig. 1:**
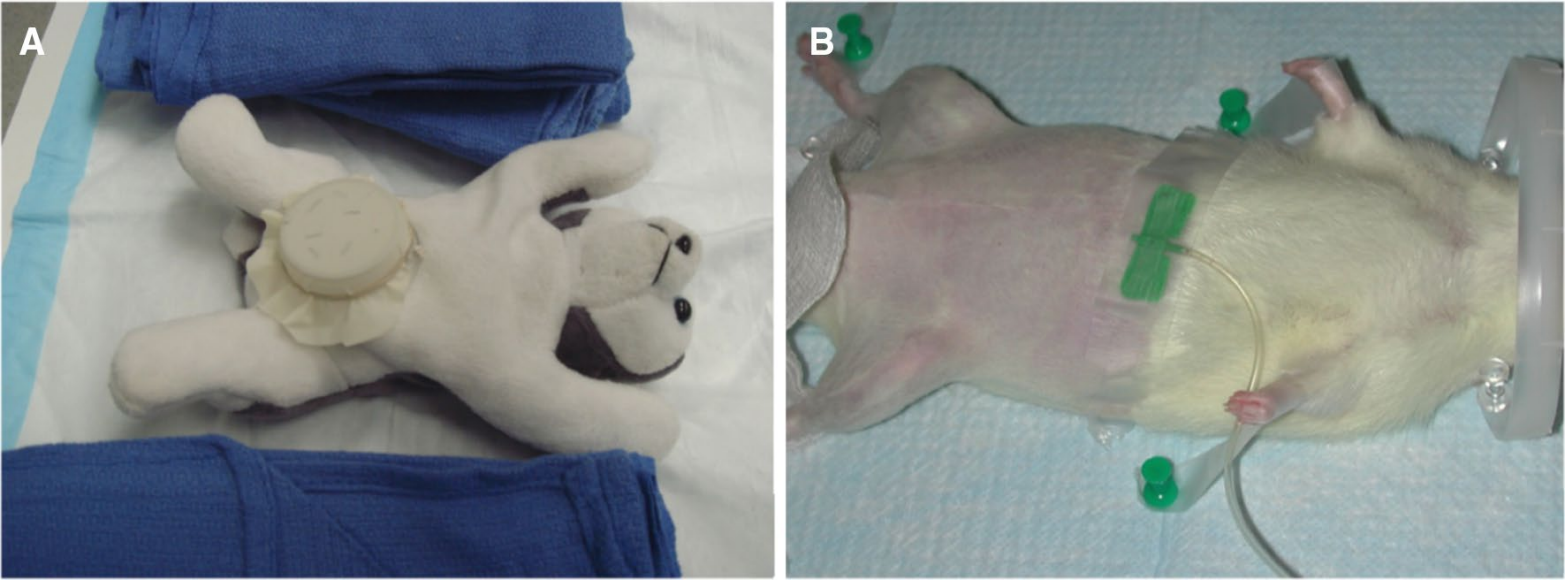
**a**: Non-live model. **b**: Preparation of the live rat

**Fig. 2:**
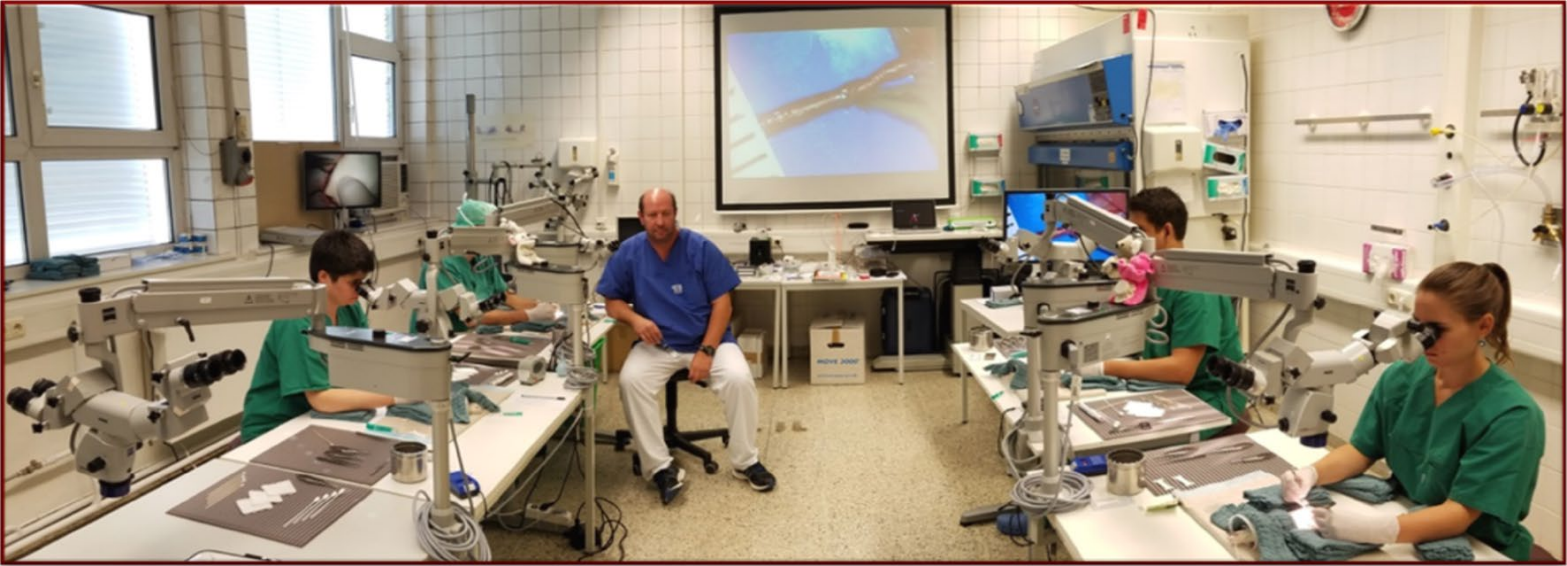
Full-time hands-on instruction

The Frankfurt Microsurgery Teaching Course, or Microcourse is an intensive 5-day, 7 h per day course that follows a curriculum created and developed by Dr. Robert D. Acland MBBS, FRCS in the mid 1970s in Louisville, USA. The course is taught throughout the year in Louisville, Dublin, Ireland, Groningen, The Netherlands, and Santiago, Chile and has been taught in Frankfurt 4–6 times per year since 2008. The course is taught in English and uses detailed and systematic, step-by-step instruction on DVD, non-live models, and live rats supported by full-time, one-on-one instruction by an experienced microsurgeon, instructor (Figs. 1a, b, 2). Throughout the 1-week-long course, all trainees begin the day viewing DVDs that focus on the specific techniques they will learn that day. They then go to the lab and practice, practice, and more practice on non-living models (day 1) and live rats (days 2–5), asking questions to the instructor and reviewing the DVDs as needed throughout the week.

The topics and techniques covered each day consist of:

Day 1: Basic posture, handling, and care of instruments, and suture exercises on a non-living practice model.

Day 2: Anastomosis of femoral artery(s) in live rats.

Day 3: Anastomosis of femoral vein(s) in live rats.

Day 4: Interpositional vein graft technique in live rats.

Day 5: End-to-side anastomosis in live rats.

Trainees who advance more quickly go on to perform advanced anastomosis techniques, not reached by all participants. These include, one-way-up and continuous-suturing-techniques. The importance of learning one skill solidly before progressing on to the next is strongly encouraged while avoiding time-wasting repetition.

The microcourse admits a maximum of only five trainees per course and its primary goal is to; “lay a solid foundation in the basic skills of microsurgical technique”. An important aspect of the course highlighted both in the instructional videos and by the instructor continuously throughout the week is “what not to do”. These “most typical mistakes of beginners” when unnoticed in the early phase of skill acquisition may lead to bad habits that are difficult to overcome later in clinical practice.

**Fig. 3:**
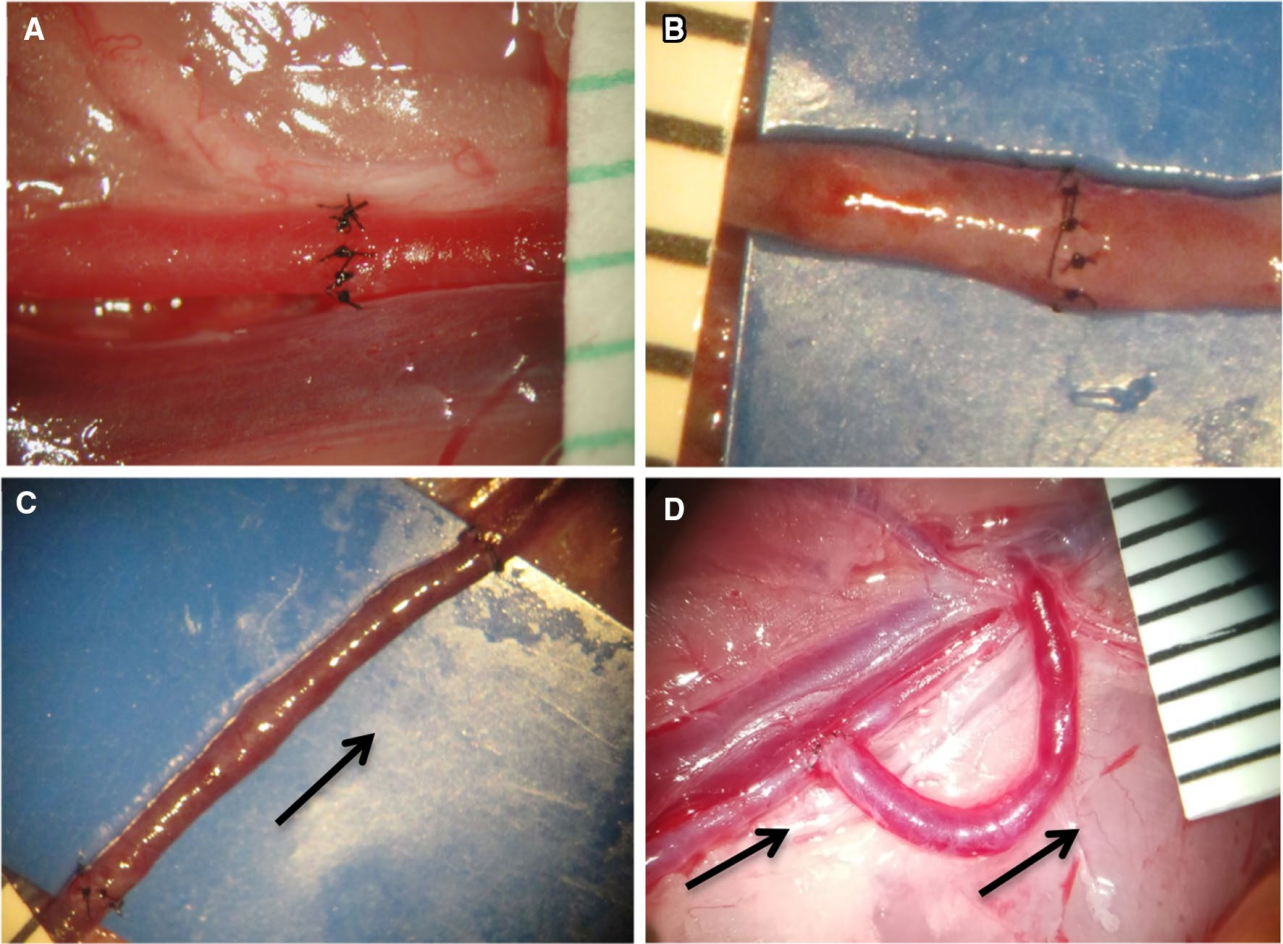
The four main vessel anastomosis techniques taught in the microcourse;** a** Arterial end-to-end anastomosis,** b** Venous end-to-end anastomosis, **c** Interpositional vein graft anastomoses, and** d** End-to-side anastomosis.* Arrows* indicate the direction of blood flow. Marks on the overlaid ruler = 1 mm

The microsurgical techniques taught are arterial anastomosis, venous anastomosis, vein graft, end-to-side technique, and optionally, one-way-up, and continuous-suturing techniques (Fig. 3). The goal of the course is that all participants acquire the necessary skills to be able to successfully perform each of these techniques at least once during the week. However, if a participant wants to practice one of the techniques more and avoid practicing others, his/her wish is respected. By the end of the course, participants should be able to perform the above techniques with: comfort and peace-of-mind, consistent use of proper hand position, efficient use of microsurgical instruments and equipment, and a disciplined, step-by-step approach to the preparatory aspects of a microsurgical procedure. Former students have indicated that in addition to helping improve their clinical microsurgical skills, the course has improved their ability to assist in clinical microsurgical cases.

Between 2008 and 2016, more than 175 trainees from several different countries and from a variety of different disciplines have taken the Frankfurt Microcourse. Here, we report data collected from these course participants from two different sources: (1) instructor’s in-course assessment of the trainee’s skill performing the anastomoses and (2) trainee’s post-course assessment of the course’ effectiveness. This represents several different aspects of the Frankfurt Microcourse, including number and patency rates of anastomoses performed, demographics of the trainees, and trainee’s impression of the usefulness of the course several months after taking the course.

## Materials and methods

### Instructors’ in-course skill assessment

**Fig. 4:**
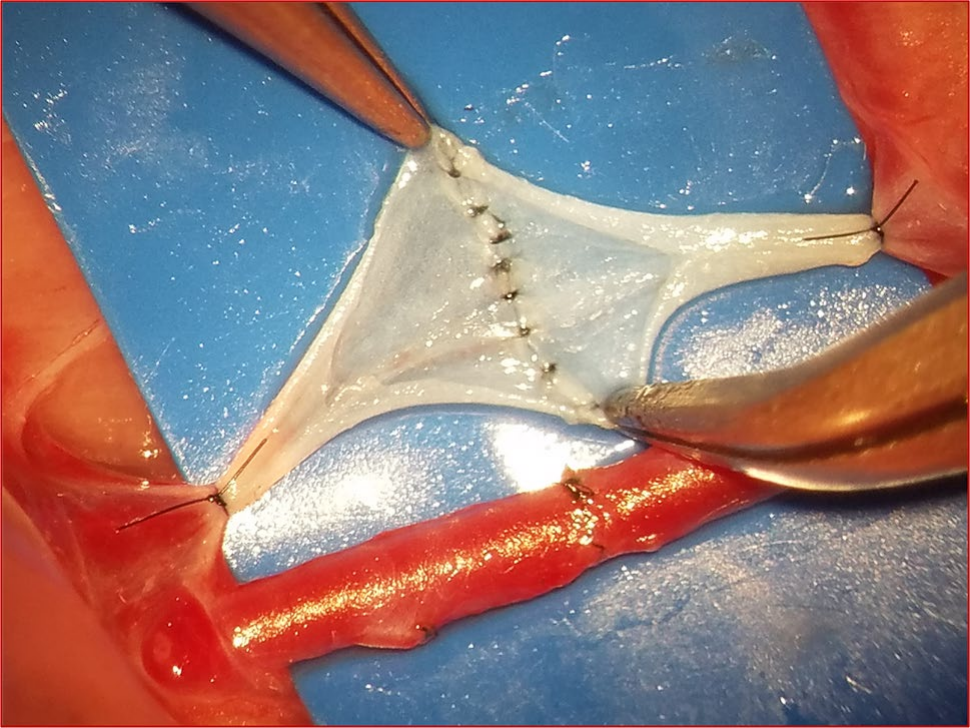
Inspection with the instructor of a venous anastomosis, on the inside of the vessel lumen to asses the quality of suturing technique

To assess and monitor trainee progress throughout the course, the instructor records the number and quality of anastomoses performed by each trainee. This is achieved by counting the number of anastomoses performed, and patency rates, after each technique (artery, vein, interpositional vein graft, and end-to-side) is completed. After performing each anastomosis and assessing patency, the instructor opens the anastomosed vessel to show the trainee how the sutures appear on the inside lumen of the vessel (Fig. 4). This allows the trainee to better assess the precision of his/her suturing technique as seen by the passing blood. In the event of thrombus formation, the trainees are shown the actual thrombus formed in the lumen at the anastomosis and its cause, i.e., poor suture technique.

### Trainee demographics

**Fig. 5:**
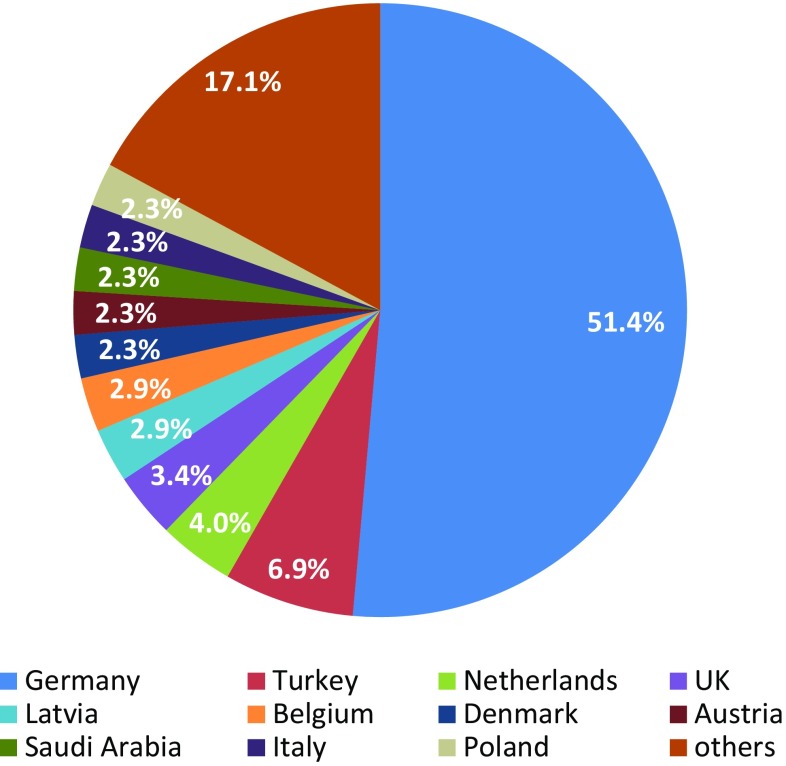
Country of origin

**Fig. 6:**
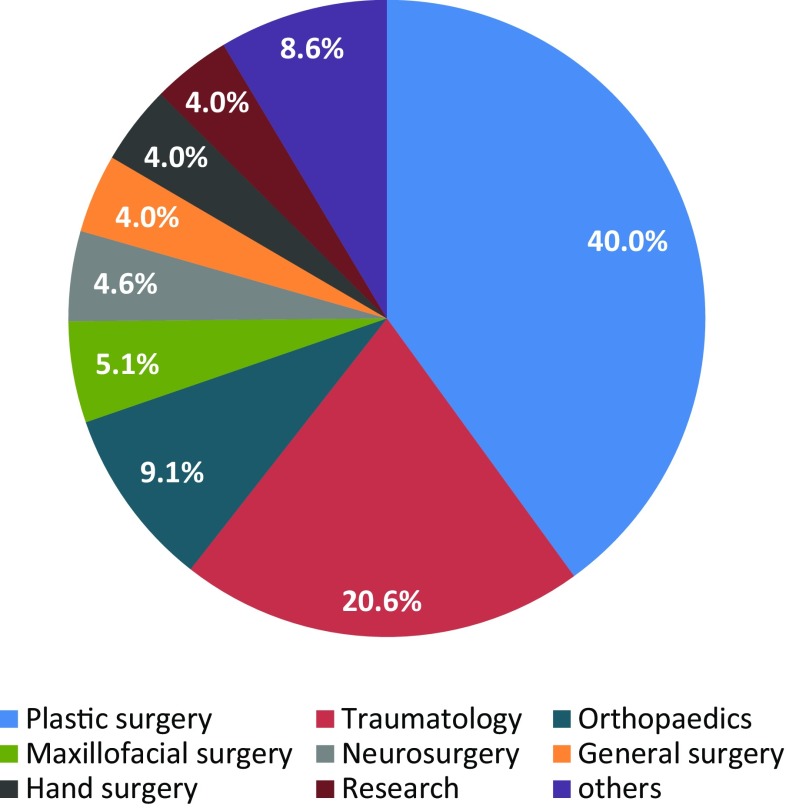
Specialty of course participants

During the course, the instructor collects basic demographic information from the participants to determine who is taking the course (Figs. 5, 6).

### Trainees’ post-course assessment of the courses’ effectiveness

Between September 2015 and March 2016, emails were sent to former course participants inviting them to participate in an online survey (SurveyMonkey software, Palo Alto, USA) that assessed their impression of how taking the course influenced their clinical microsurgical practice and the quality of the course using the ten questions, listed in Table 1.

**Table 1:** Post-course online survey questions

Current level of surgical training? Level of microsurgical skills prior to and after taking the course? Number of microsurgical cases performed per year before and after taking the course? Pace of the instruction during the course? Did the course improve your clinical microsurgical skills? After taking the course do you feel more comfortable doing clinical microsurgical cases? Would you benefit from taking a refresher course? What aspect of the microsurgery course was most valuable? Would you recommend the course to a colleague? In which clinical area did the skills you learned in the course help you most?	

## Results

### Trainee demographics

Trainee gender, age, and previous microsurgery experience are presented in Table 2. Fifty-three of 175 trainees were female, and 122 were male. Their mean age was 34.5 years, with the youngest being 25 and the oldest 70 years. Twenty-one course participants had previously attended a microsurgery course, while 154 had not. Forty three participants had no previous clinical experience in microsurgery, 77 had less than 1 year of experience, 36 had between 1 and 2 years, and 19 had more than 2 years of experience (Table 2). Course participants hailed from 32 countries (Fig. 5), and were from the following subspecialties, in descending order, 70 were from plastic and reconstructive surgery, 36 from traumatology, 16 from orthopedics, 9 from maxillofacial surgery, 8 from neurosurgery, 7 from general surgery, and 7 from hand surgery, 7 were research fellows, and 15 were from other specialties (Fig. 6).

**Table 2:** Gender, age, and previous microsurgery experience of course participants

	*n* (%)	Mean (SD)
Female	53 (30)	
Male	122 (70)	
Age (years)		34.5 (6.4)
Previous microsurgery course		
Participants who had taken a microsurgery course previously	21 (12)	
Participants who had not taken a microsurgery course previously	154 (88)	
Previous clinical microsurgery experience		
No experience	43 (24.6)	
Less than 1 year	77 (44.0)	
Between 1 and 2 years	36 (20.6)	
More than 2 years	19 (10.9)	

### In-course assessment of trainee anastomoses skills: number and patency rate of anastomoses

During the course 94.8% (165 of 174) of the participants performed at least one patent arterial anastomosis, 59.9% (100 of 167) performed at least one patent venous anastomosis, 54.7% (81 of 148) performed at least one successful vein graft, 85.4% (135 of 158) performed at least one patent end-to-side anastomosis, 96.1% (49 of 51) performed at least one successful arterial anastomosis using one-way-up technique, 57.1% (12 of 21) performed at least one successful venous anastomosis using one-way-up technique, and 90.9% (10 of 11) performed at least one patent anastomosis using continuous-suture technique (Table 3). The total number of trainees that performed each of the different anastomosis techniques (represented by the second number in parenthesis) differs in each case, because for different reasons (preferred to focus on another technique, did not feel sufficiently competent to perform the given technique, etc), not all participants attempted all the techniques during the 5-day course.

**Table 3:** Number and patency rates of different anastomoses performed

	No of participants who attempted a given task at least once	No of participants who performed at least one patent anastomosis	% of participants who performed at least one patent anastomosis	No of overall attempts at a given task	No of patent anastomoses overall	% of successfully performed tasks
Arterial anastomosis	174	164	**94.8**	436	356	**81.7**
Venous anastomosis	167	100	**59.9**	319	141	**44.2**
Venous graft	148	81	**54.7**	166	85	**51.2**
End-to-side anastomosis	158	135	**85.4**	180	148	**82.2**
One-way-up artery	51	49	**96.1**	56	54	**96.4**
One-way-up vein	21	12	**57.1**	22	12	**54.5**
Continuous-suture technique	11	10	**90.9**	11	10	**90.9**

The overall patency rates for arterial anastomoses was 81.7%, for venous anastomoses 44.2%, for venous graft anastomoses 51.2%, for end-to-side anastomoses 82.2%, for arterial anastomoses using one-way-up technique 96.1%, for vein anastomoses using one-way-up technique 54.5%, and for arterial anastomoses using continuous-suture technique 90.9% (Table 3).

### Post-course trainee’ assessment of the courses’ effectiveness

Of the emails sent to former participants requesting that they complete the online survey, only 42 responded. At the time, they completed the survey 78.6% were residents and 21.4% were in practice. While 85.7% of respondents “agreed” or “strongly agreed” that they would benefit from taking a refresher course, all (100%) responded that their microsurgical skills had improved significantly after taking the course and that they are now more comfortable performing clinical microsurgery cases.

**Fig. 7:**
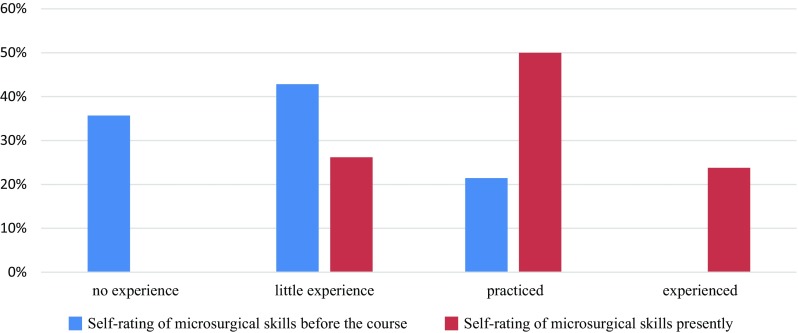
Microsurgical experience at the time of the course and presently

**Fig. 8:**
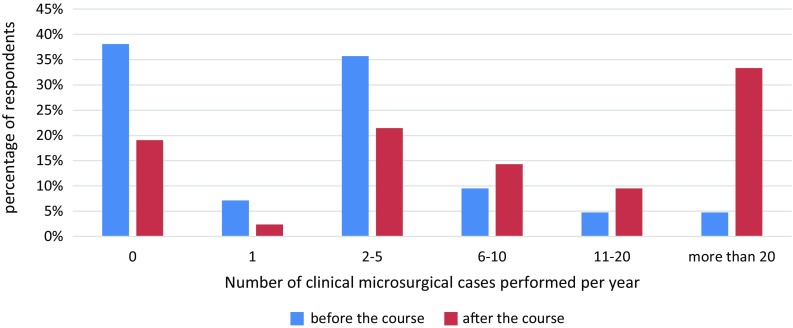
Number of microsurgical cases performed per year before the course and presently

On a four-point scale from “not experienced” to “experienced”, none of the respondents considered themselves to be “experienced” at the time they took the microcourse (35.7% had no experience, 42.9% little experience, and 21.4% were practiced). In contrast, at the time, they filled out the survey (several months after taking the course) 23.8% of the respondents considered themselves to be “experienced”, 50% “practiced”, and 26.2% still had “little experience” (Fig. 7). The number of clinical microsurgical cases performed by course participants after taking the course increased considerably. At the time, they took the microsurgery course only 4.8% of trainees reported having performed more than 20 cases per year, while when they completed the survey, this figure had increased to 33% (Fig. 8). Furthermore, former participants indicated that the course had helped them the most to perform microvascular anastomosis (83.3%), followed by tissue handling (60.5%) and finally nerve anastomoses (47.6%).

When asked the “most” to the “least” valuable part of the course 66.7% of participants responded that the one-on-one instruction by the full-time instructor was the most, and 71.4% considered the course workbook to be the least valuable. All 100% of the former trainees answered that they would recommend the course to a colleague.

## Discussion

Microsurgery is performed in many subspecialties. In some, such as Plastic Surgery and Hand Surgery, it is well established and widely used, while in others, it has a supplementary role. The prevalence of microsurgery in the respective subspecialties was reflected in this course with plastic surgeons making up 40% of all course participants, followed by trauma surgeons (20.2%) and orthopedics (9.1%) (Fig. 6). This relatively high representation of trauma surgery residents is due to the fact that many residents in the Department of Trauma, Hand, and Reconstructive Surgery in Frankfurt, where the course is held, attend the course. Hand injuries represent one-third of the patients admitted to trauma emergency departments; therefore, the microsurgery skills and soft tissue handling taught in the microcourse are valuable and useful skills in their daily work.

Simulation training, outside the operating theatre, is becoming an important method to practice microsurgery technique for many reasons, with patient safety and reduction in resident training time being the most important [12, 13]. Considering the steep learning curve in microsurgery, simulated training may help to overcome some of the initial difficulties in acquiring the necessary skills [14].

During 1-week-long microsurgery courses, participants must learn several highly refined manual skills often for the first time and in a relatively short amount of time. Hours of concentrated work under the microscope are strenuous and although the results, especially performing venous anastomoses, can be challenging participants do gain a solid foundation in the basic skills of microsurgical technique. With this foundation acquired in a microsurgery course as a first step, it is essential that following the course trainees go on and apply these skills in clinical practice.

In a report of the consensus workshop on microsurgical training at the 32nd Annual Meeting of the German-Language Group for Microsurgery of Nerves and Vessels (DAM), a draft of a standardized microsurgery training curriculum was presented. This draft contained a “basic module”, whose content is similar to many of the microsurgery courses offered in Germany and internationally [15]. The basic module includes tasks that once completed should enable a trainee to perform microsurgical operations, of low level of difficulty, such as arterial anastomoses, on patients. The results of our course show that achieving this level of skill is realistic as demonstrated by the 94.8% of participants that were able to perform at least one patent arterial anastomosis during the course. This was confirmed in the post-course survey in which most respondents indicated that their microsurgical skills had improved as a result of taking the course and that they are now more comfortable performing clinical microsurgery cases. This is further supported in their post-course responses in which they indicated that prior to taking the course 0% were "experienced", 21.4% were "practiced", 42.9% had "little experience", and 35.7% had "no experience". In contrast, after taking the course, 23.8% reported being “experienced”, 50% “practiced”, and 26.2% with “little experience”. This is further accentuated in the important increase in the number of clinical microsurgery cases performed by course participants, prior to (4.8% of trainees reported performing >20 cases/year) and after (33% reported performing >20 cases/year) taking the course.

Our data show that there is a clear difference in the level of difficulty of the different microsurgical techniques taught. While 94.8% of participants were able to perform at least one patent arterial anastomosis, only 59.9% were able to perform a patent venous anastomosis. Overall patency rates for arterial anastomoses were 81.7%, while patency rates for venous anastomoses were only 44.2%. Patency rates of venous grafts were similar to those of venous anastomoses where almost 54.7% of participants performed at least one patent venous graft, while the overall success rate of this task was 51.2%. End-to-side anastomoses were successfully performed at least once by 85.4% of participants, with overall patency rates being 82.2%.

These numbers clearly indicate that venous anastomoses are considerably more challenging to perform than arterial anastomoses. Both trainees with and without previous experience have difficulty with vein anastomoses. In the rat model, the initial dissection of the vein is key to achieving patency. If the vein is traumatized during the dissection, even a little; thrombosis is very likely to occur. It is almost like you must dissect the vein “without touching it”. This observation is emphasized in a report by Hui KC, et al. in which the authors claim that “25–30 venous anastomoses are necessary for a beginner to reach patency rates comparable with experienced microsurgeons” [16].

Interestingly, participants that chose to perform the additional, and arguably more difficult “optional” tasks—arterial anastomosis using one-way-up technique and the continuous-suture-technique—achieved notably high patency rates of 96.4 and 90.9%, respectively. An explanation for this is that trainees with higher levels of skill were the ones who typically chose to perform these optional tasks. Of note is that trainees that performed the one-way-up technique in veins only achieved patency rates of 54.5%, which were comparable to rates achieved by all participants when they performed the standard end-to-end venous anastomoses (44.2%). This suggests that this task was challenging even for the more experienced trainees and suggests that a refresher course designed to improve venous anastomoses skills could be valuable.

One-way-up technique is a very elegant way to suture a vessel without turning over the clamp. Trainees are taught to suture the vessel from the back to the front. The main trick to accomplish this task is that a stay suture must be fixed to the frame of the double clamp. This anastomosis is learned without difficulty and with a little experience, the time to complete it can be reduced significantly. The continuous-suture anastomosis is very challenging, especially in 1 mm diameter vessels. The instructor has noticed over the years that the most problematic aspect of performing this technique is making sure that the diameter of the vessel is maintained and not allowed to narrow. To avoid this, trainees are taught to perform this technique using two separate continuous sutures: one for the anterior wall and the second for the posterior wall. In this way, there is less chance of reducing and narrowing the vessel lumen.

The primary reason for recording the number of completed anastomoses and patency rates throughout the week is to monitor the progress of individual students and to continuously assess and improve the quality of our teaching methods. While anastomotic patency is essential to success in clinical microsurgery, other methods have been developed and are used to assess microsurgical skill acquisition. One such method called Likert scales is based on global rating scales [17–19]. This scale has a rating from 1 to 5 and consists of a set of specific aspects of the anastomosis technique that are considered to be essential for microsurgical patency. For example, the “quality of knot”, whereby a score of 1 corresponds to “not square, loose, cut ends too long/short”, while a score of 3 corresponds to “partially square, somewhat loose, cut ends OK length” and a score of 5 corresponds to “square, snug, cut ends, and proper length” [4]. Another global rating scale called the “Objective Structured Assessment of Technical Skills (OSATS)” assesses surgical performance. We used a modified version of this rating method to assess skill improvement over time in our Frankfurt Microcourse. During a few select week-long courses, we applied the revised OSATS method on day 2, and then again on day 5. The results showed a significant improvement in the OSATS scores as well as a significant reduction of the time necessary to perform the anastomosis. From these studies, we were able to conclude that “participating in a microsurgical training course results in significant improvement in objectively assessed microvascular surgical skills. The degree of skill improvement was strongly correlated with psychomotor aptitude assessments scores for trainees” [20]. While these assessment tools can provide useful information about a trainees’ progress and can help to assess the effectiveness of teaching methods, we have found that they are somewhat cumbersome to implement on an ongoing basis and tend to distract trainees from their learning experience so we only use them on occasion.

When asked in both the in-course questionnaire and in the post-course online survey “what was the most valuable part of the course”, most participants considered the one-on-one instruction provided by the full-time instructor. The importance of having an experienced instructor present full time has also been emphasized by residents and plastic surgery programs directors [11].

While most of the respondents stated that the course helped them most in performing microvascular “anastomoses” and in “tissue handling”, almost half also report improvement in their ability to perform nerve coaptations, even though this task is not taught in the Frankfurt Microcourse. Perhaps, improved handling of instruments and “feel” for the tissue contributed to this response.

## Conclusion

The fact that the majority of course participants were able to successfully perform arterial end-to-end anastomoses suggests that it is realistic for a beginner to reach a level of proficiency to be able to perform similar tasks on patients, under supervision. In contrast, the lower patency rates achieved by trainees when performing venous anastomoses or venous grafting suggest that this task might require further practice. Overall, all respondents felt that their microsurgery skills improved significantly after attending the course. One of the most important aspects of a good microsurgery course is the full-time presence of a knowledgeable and experienced instructor.
